# Synthesis, Photophysical Characterization, and Anticancer Evaluation of Novel Furochromene Derivatives

**DOI:** 10.1002/open.70213

**Published:** 2026-05-21

**Authors:** Amal Rabahi, Mustapha Mounir Bouhenna, Hasnia Abdeldjebar, Massinissa Baymout, Malika Makhloufi‐Chebli, Walter Luyten, Hamdi Bendif, Anis Ahmad Chaudhary, Walid Elfalleh, Stefania Garzoli

**Affiliations:** ^1^ Applied Organic Chemistry Laboratory Faculty of Chemistry University of Sciences and Technology Houari Boumediene Algiers Algeria; ^2^ Centre de Recherche Scientifique et Technique en Analyses Physico‐chimiques (CRAPC) Tipaza Algeria; ^3^ Laboratory of Physics and Chemistry of Materials (LPCM) Department of Chemistry Faculty of Sciences Mouloud Mammeri University Tizi‐Ouzou Algeria; ^4^ Department of Biology Animal Physiology and Neurobiology Section Leuven Belgium; ^5^ Department of Biology College of Science Imam Mohammad Ibn Saud Islamic University (IMSIU) Riyadh Saudi Arabia; ^6^ Department of Chemistry and Technologies of Drug Sapienza University Rome Italy

## Abstract

A series of novel furochromen derivatives, namely (*2E*)‐1‐(6‐hydroxy‐4,7‐dimethoxy‐1‐benzofuran‐5‐yl)‐3‐arylprop‐2‐en‐1‐ones **4a–c**, were synthesized via the Knoevenagel condensation of 6‐hydroxy‐4,7‐dimethoxybenzofuran‐5‐ylethanone **2** with various aromatic aldehydes. Molecular structures were fully established using ultraviolet–visible spectroscopy (UV–vis), fourier transform infrared (FTIR), Mass Spectrometry, and nuclear magnetic resonance (NMR) techniques. The photophysical characteristics of the compounds were systematically studied in various solvents at 298 K., revealing significant solvatochromic behavior and fluorescence emission, suggesting their potential as fluorescent probes for biological imaging. In addition, quantum chemical calculations discrete fourier transform (DFT)/B3LYP) were performed to determine highest occupied molecular orbital‐lowest unoccupied molecular orbital (HOMO–LUMO) energies and dipole moments in both ground and excited states. To assess their biological relevance, the synthesized compounds were tested for their antiproliferative activity against human cancer cell lines A549 (lung), HepG2 (liver), and HCT‐116 (colon), using real‐time cell impedance spectroscopy. Among the tested molecules, compound 4c showed the most potent cytotoxic effect, with complete inhibition of cell viability in A549 and HepG2 cells and an IC50 of 4.6 µM against A549. These findings highlight the dual potential of furochromene derivatives as both photophysical tools and promising anticancer candidates.

## Graphical abstract

This is the first study on novel furochromene derivatives.

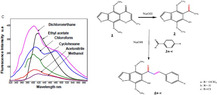

## 1 Introduction

Cancer is the second leading cause of death in the world, following cardiovascular disease. The World Health Organization (WHO) predicts that cancer will be responsible for 12 million deaths globally by 2030 [1]. Notably, lung cancer is among the most common types of cancer in terms of incidence and ranks within the top five causes of cancer‐related mortality [2].

Cancer is the second leading cause of death worldwide, after cardiovascular diseases. The World Health Organization (WHO).

**FIGURE 1:**
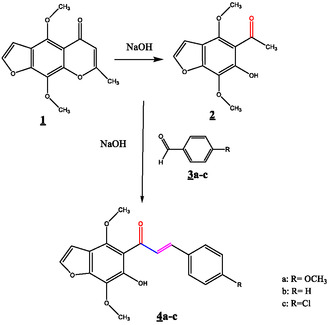
Schematic representation of the synthesis of compounds **4a‐c** .

The deployment of many anticancer drugs is associated with intense toxicity as a result of their mechanisms of action and nonspecificity. The persistence of resistance to chemotherapy drugs continues to hinder cancer therapy; therefore, there is vast scientific attention on the discovery of new anticancer drugs. The deployment of many anticancer drugs is associated with intense toxicity as a result of their mechanisms of action and lack of specificity. The persistence of resistance to chemotherapy drugs continues to hinder cancer therapy; therefore, there is significant scientific interest in the discovery of new anticancer agents. In addition to developing new anticancer agents to improve treatment efficacy and reduce toxicity, it is also necessary to identify single molecules containing more than one pharmacophore for effective cancer treatment [3, 4, 5]. The choice of heterocyclic fragments is important in medicinal chemistry [6, 7, 8, 9, 10]. Among all heterocyclics, khellin derivatives or fused furochromene are branded bioactive agents. Various natural derivatives—compounds of furochromene—have been reported as anticancer agents [7]. They are favorable in the development of antitumor agents with the potential to inhibit cell proliferation in multiple carcinoma cell lines [11, 12]. Moreover, it has been indicated that this heterocyclic family has various biological applications, such as antiviral, antifungal, antibacterial, antidiabetic, neuroprotective, and anti‐inflammatory activities [13]; it has been applied in the photochemotherapeutic treatment of asthma [11] and psoriasis [14, 15, 16]. Also, it is used as a vasodilator and antispasmodic [17]; furochromenes are used for their high lipid‐altering, antiatherosclerotic, and analgesic activities, and they represent the primary active component of many medicines [18]. They are also used for bronchial asthma and vitiligo treatment [19]. Several reports indicate that furanochromones, especially furochromene derivatives, demonstrate notable inhibitory effects on epidermal growth factor receptor (EGFR) and vascular endothelial growth factor receptor (VEGFR) [6, 20]. All potential uses of the furochromene class prompted us to undertake and develop our research in this area in order to synthesize molecules that may exhibit properties similar to those mentioned previously. One of the most widely employed methods for the preparation of ketones and α,β‐unsaturated ester derivatives from furochromene is the Knoevenagel condensation (Figure 1). With the aim of synthesizing more efficient fluorescent compounds, Lippert's, Bakhshiev's, and Kawski–Chamma–Viallet equations are usually used to investigate their photophysical behavior [21]. Fluorescence makes it possible to highlight the cells attacked by a tumor, locate it, and observe its evolution [22]. The importance of furochromene and its derivatives prompted us to undertake a computational study, which is increasingly establishing a reliable method for modeling certain chemical phenomena where the energy of an electronic system is determined [21, 23]. In this study, we synthesized a series of furochromene derivatives and comprehensively evaluated their physicochemical and biological properties. Their cytotoxic potential was assessed against three human cancer cell lines: A549 (lung), HepG2 (liver), and HCT‐116 (colon), using real‐time cell viability monitoring based on electrical impedance spectroscopy. The optical properties of these compounds were examined through UV‐Vis and fluorescence spectroscopy in various solvents to investigate their solvatochromic behavior and fluorescence emission capacity. Theoretical calculations, including dipole moment analysis and Highest Occupied Molecular Orbital‐Lowest Unoccupied Molecular Orbital (HOMO–LUMO) energy gap determination, were performed to gain deeper insight into their electronic structure and potential interactions with biological targets. Taken together, these multidisciplinary approaches aim to highlight the dual role of furochromene‐based molecules as both fluorescent probes and promising anticancer agents.

## 2 Material and Methods

### 2.1 Apparatus

Melting points were determined by the Electrothermal Stuart Scientific SPM3 apparatus fitted with a microscope and are uncorrected. IR spectra (KBr) were recorded on an FT‐IR Perkin Elmer (Spectrum One). ^1^H and ^13^C spectra were recorded in deuteriochloroform solutions and DMSO‐d_6_ with TMS as internal standard on a BRUKER Avance 300, carbon: 75.47 MHZ, proton: 300.13 MHZ; the chemical shifts are expressed in δ (ppm).

All spectrophotometric recordings were performed at ambient temperature. UV/Vis absorption spectra were measured on a Varian CARY 50 conc. The approximated experimental error was 2 nm on the band maximum and 5% on the molar extinction coefficient. Fluorescence studies were conducted on a Varian CARY Eclipse spectrofluorometer. All excitation and emission spectra corrected. The fluorescence quantum yields were evaluated and determined using fluorescein disodium salt (Φ = 0.9) as standard.

The fluorescence quantum yields (Φ) were determined using the classical formula:




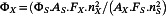




Where “*A*” is absorbance at the excitation wavelength, “*F*” the area under the fluorescence curve, and “*n*” is the refractive index of the solvents used. Subscripts “*s*” and “*x*” refer to the standard and to the sample of unknown quantum yield, respectively. The excitation source was a long‐life Xenon flash lamp.

### 2.2 General Procedure for the Synthesis of (*2E*)‐1‐(6‐Hydroxy‐4,7‐Diméthoxy‐1‐Benzofuran‐5‐Yl)‐3‐(aryl) Prop‐2‐en‐1‐One 4a‐c

4,9‐dimethoxy‐7‐methyl‐5*H*‐furo[3,2g]benzopyran‐5‐one **1** (2.6 g, 10 mmol) was added to 1g of NaOH in 100 mL of distilled water. The mixture is heated for 1 h, and the final solution is cooled in a bath of cold water. The solution's pH was carefully monitored and maintained between 6 and 7 using a calibrated pH meter while gradually adding 1N HCl to prevent sudden acidification. The yellow crystalline precipitate appeared when the pH dropped to 3–6, indicating that crystallization occurs under slightly acidic conditions. Precise pH control is essential to optimize the yield and maintain the quality of the product 2. Compound **2** was then washed with cold distilled water and recrystallized from methanol.

Yellow solid (1.89g, 80%); mp 101°C‐103°C; IR: (ν) 3135, 1623, 1540, 1463, 1380, 758 cm^−1^; ^1^H NMR (CDCl_3_): δ 2.72 (s, 3H, H‐CO(CH_3_)), 4.03 (s, 3H, H‐OCH_3_), 4.14 (s, 3H, H‐OCH_3_), 6.89 (d, 1H, H‐3, *J* = 2.7 Hz), 7.48 (d, 1H, H‐2), 13.14 (s, 1H, H‐6); ^13^C NMR (CDCl_3_): δ33.71 (CH_3_), 60.93, 61.43 (OCH_3_), 110.84 (C3a), 111.13 (C5), 129.08 (C7), 144 (C2), 151 (C6), 152.6 (C7a), 153.80 (C4), 205.55 (C = O); ESI(+)‐MS: m/z 237.001 [(M + H)^+^, 15], 259.001 [(M + Na)^+^, 30], 459.008 [(2M+Na)^+^, 100].

#### 2.2.1 Synthesis of Compound (2E)‐1‐(6‐Hydroxy‐4,7‐Diméthoxy‐1‐Benzofuran‐5‐yl)‐3‐(aryl) Prop‐2‐en‐1‐One 4a‐c

5‐acetyl‐6‐hydroxy‐4,7‐dimethoxybenzofuranne **2 (**40 mmol) was added to a stirring solution of arylaldehyde (40 mmol) in 10 ml of ethanol with 2 ml of 10% aqueous NaOH. The mixture was stirred for 7 h at room temperature. The solution was neutralized with 2 ml HCl 10%. The formed solid was filtered off and recrystallized from the appropriate solvent to obtain (2E) (6‐hydroxy‐4,7‐diméthoxy‐1‐benzofuran‐5‐yl)‐3‐(aryl) prop‐2‐en‐1‐one **4a‐c**.

#### 2.2.2 (2E)‐1‐(6‐Hydroxy‐4,7‐Dimethoxy‐1‐Benzofuran‐5‐yl)‐3‐(4‐Methoxyphenyl)Prop‐2‐en‐1‐One 4a

Orange solid (0.61 g, 70%); mp 138°C‐140°C; IR : (ν) 3116, 1623, 1559,1527,1290 Cm^−1^; ^1^H NMR (CDCl_3_): δ 3.903 (s, 3H, H‐OCH_3_), 3.913 (s, 3H, H‐OCH_3_), 4.039 (s, 3H, H‐ OCH_3_), 6.95 (d, 1H, H‐3, *J* = 2.1 Hz), 7.05 (d, 2H, H‐9′, H‐10, *J* = 15.91 Hz), 7.21 (m, 2H, H‐12, H‐6′’), 7.64 (d, 2H, H‐14, H‐15, *J* = 9.3 Hz), 7.89 (d, 1H, H‐2); 9.98 (s, 1H, H‐6); ^13^C NMR (CDCl_3_): δ 55.84, 6142, 62.41 (OCH_3_), 105.6 (C3), 106.14(C3a), 112.29 (11),113.18 (C5), 114.83 (C9), 114.21 (C7), 124.78 (C11), 128.15 (C15), 129.33 (C12), 130.69 (C16),) 144.08 (C10), 144.44 (C2), 150.95 (C14), 152.00 (C4), 153.53 (C7a), 162.01 (C6), 194.84 (C8); MS‐EI (GC–MS): m/z 354 (30%), 220 (100%), 205 (79%), 177 (50%), 134 (22%), 163 (18%), 191 (10%). Anal. Calcd. for C_21_H_20_O_5_: C 71.6; H 5.7%; Found: C 71.5; H 5.6%.

#### 2.2.3 (2E) (6‐Hydroxy‐4,7‐Dimethoxy‐1‐Benzofuran‐5‐Yl)‐3‐Phenyl Prop‐2‐en‐1‐One 4b

Orange solid (0.56 g, 70%); mp127°C‐129°C; IR : (ν) 3135, 1623, 1553, 1490,1380 Cm^−1^; ^1^H NMR (CDCl_3_): 4.04 (s, 3H, H‐OCH_3_), 4.08 (s, 3H, H‐OCH_3_), 6.87 (d, 1H, H‐3, *J* = 2.1 Hz), 7.41–7.43 (m, 3H, H‐13, H‐14, H‐15), 7.52 (d, 1H, H‐2, 7.63–7.66 (m, 2H, H‐12, H‐16), 7.87 (d, 1H, H‐9, H‐10); 12.75 (s, 1H, H‐6); ^13^C NMR (CDCl_3_): δ60.93, 61.85 (OCH_3_), 105.14 (C3), 111.65 (C9), 112.55 (C3a), 126.78 (C5), 128.29 (C14), 128.35 (C15), 128.85 (C13), 129.30 (C16), 130.37 (C11), 134.92 (C2), 135.12 (C12), 143.34 (C7), 144.01 (C10), 150.59 (C7a), 151.74 (C6), 153.04 (C4), 194.54 (C8); MS‐EI (GC–MS): m/z 324 (40%), 220 (75%),205 (74%), 177 (53%) 163 (22%), 191(10%); MS (ESI+): m/z 325.001 [(M + H)^+^, 100], 347.001 [(M + Na)^+^, 11], 671.002 [(2M+Na)^+^, 60]. Anal.Calcd. for C_20_H_18_O_4_: C 71.5; H 5.6%. Found: C 71.4; H 5.5%.

#### 2.2.4 (2E)‐1‐(6‐hydroxy‐4,7‐dimethoxy‐1‐benzofuran‐5‐yl)‐3‐(4‐chlorophenyl)prop‐2‐en‐1‐one 4c

Orange solid (0.63 g, 70%); mp150°C‐152°C; IR : (ν) 3128, 1636, 1546, 1296 Cm^−1^ ; ^1^H NMR (CDCl_3_): *δ* = 4.03 (s, 3H, H‐OCH_3_), 4.06 (s, 3H, H‐OCH_3_), 6.87(d, 1H, H‐3, *J* = 2.3 Hz), 7.730–7.860 (m, 4H‐phenyl), 7.52 (m, 2H, H‐9, H‐10), 7.38 (d, 1H, H‐2), 12.7 (s, 1H, H‐6); ^13^C NMR (CDCl_3_): *δ* = 60.68, 61.75 (OCH_3_), 105.14 (C3), 111.48 (C3a), 112.31 (C5), 127.20(C9), 127.28 (C15), 129.07 (C13), 129.22 C7), 129.42 (C12), 133.40 (C16), 136.12 (C14), 141.64(C11), 144.00 (C10), 144.20 (C2), 150.55 (C7a), 151.80 (C6), 152.99 (C4), 194.23 (C8). MS‐EI (GC–MS): m/z 358 (21%), 220 (86%), 205 (72%), 177 (47%), 138 (11%), 163 (16%), 191 (9%). Anal. Calcd. for C_20_H_17_ClO_4_: C 67.3; H 4.8; Cl 9.9% Found: C 67.2; H 4.9; Cl 10.0; %.

### 2.3 Cytotoxic Evaluation

#### 2.3.1 Cell Culture

The mammalian cell lines used in this study were the human colorectal carcinoma cell line HCT‐116 (RRID: CVCL_0291; ECACC 91 091 005), human hepatocellular carcinoma cell line HepG2 (RRID: CVCL_0027; ECACC 85 011 430), and human lung adenocarcinoma epithelial cell line A549 (RRID: CVCL_0023; ECACC 86 012 804). All adherent human cell lines were obtained from the European Collection of Authenticated Cell Cultures (ECACC, Salisbury, UK). HepG2 and A549 cells were cultured in Dulbecco's Modified Eagle's Medium (DMEM) supplemented with 10% fetal bovine serum (FBS) and 1% penicillin/streptomycin (P/S). HCT‐116 cells were cultured in McCoy's 5A medium supplemented with 10% FBS and 1% P/S. All cells were maintained under standard conditions in a humidified atmosphere at 37°C with 5% CO_2_. Cells were routinely subcultured two to three times per week.

#### 2.3.2 Monitoring of Cellular Viability

Cell proliferation was continuously monitored for 48 h using an RT‐CES from *Acea* Biosciences System as described previously [24]. 24 h after seeding, test compounds were added to the cells with five different concentrations (100, 70, 50, 35, and 10 µM) for 24 h. Three replicates on one 96‐well plate were performed.

Impedance measurements were recorded every 20 min following compound administration and continued until the end of the experiment. To ensure consistency, the data from each well were normalized to the first measurement taken after treatment initiation, according to the formula: normalized |Z| =|Z| at time x/|Z| at reference time. These normalized impedance values were used for graphical representation and subsequently exported to MATLAB for further analysis.

### 2.4 Statistical Analysis

Statistical analyses were performed for the cell viability assays using GraphPad Prism version 9.5.1 (733) (GraphPad Software, San Diego, CA, USA).

Data are presented as mean ± standard deviation (SD) from three independent experiments (*n* = 3 per condition). No data transformation or normalization was applied. Outliers were assessed visually, and no values were excluded.

Comparisons among multiple treatment groups were performed using one‐way analysis of variance (ANOVA), followed by Tukey's or Dunnett's post hoc tests, as appropriate. Tukey's test was applied for pairwise comparisons among compounds, and Dunnett's test was used when each treatment group was compared directly to the reference control (gossypol). All tests were two‐sided, with a significance threshold of *α* = 0.05. Adjusted *p* values were calculated using the respective post hoc corrections to account for multiple comparisons.

Prior to ANOVA, assumptions of normality of residuals and homogeneity of variances were evaluated. Statistical significance in figures and tables is indicated as follows: **ns**, not significant; *p < 0.05; **p < 0.01; ***p < 0.001; ****p < 0.0001.

## 3 Results and Discussion

### 3.1 UV–visible Absorption Analysis

The electronic absorption spectra of compounds **4a–c** (10^−5^ M) were recorded immediately after dissolution and filtration to ensure spectral stability and reproducibility. The observed Stokes shifts range from 3234 to 12 898 cm^−1^, indicating significant excited‐state reorganization and suggesting the presence of intramolecular charge transfer (ICT) character.

**FIGURE 2:**
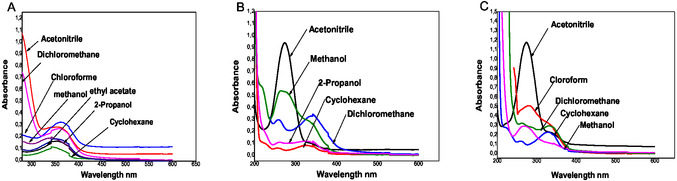
(A–C) Absorption spectra of compounds (A) **4a**, (B) **4b**, (C) **4c** in various solvents.

In acetonitrile, methanol, ethylacetate, chloroform, 2‐propanol, dichloromethane, and cyclohexane, the absorption spectra exhibit two principal absorption regions (Figure 2a–c). The intense band observed in the 320–360 nm region is attributed to an allowed π→π* transition involving the extended conjugated benzofuran–chalcone framework. The relatively high molar extinction coefficients (*ε* = 1.0 × 10^4^ to 1.18 × 10^5^ M^−1 ^cm^−1^) support this assignment.

A weaker n→π* transition, expected to arise from the nonbonding electrons of the carbonyl oxygen, is not distinctly resolved in the recorded spectra and appears to be overlapped by the dominant π→π* band, resulting in a broadened absorption envelope centered around 300–350 nm. The broad profile of this band suggests partial superposition of electronic transitions, possibly enhanced by vibronic contributions and solvent–solute interactions.

Frontier molecular orbital (FMO) analysis performed at the DFT/B3LYP/6‐311G(d, p) level provides qualitative support for this interpretation. The HOMO is mainly localized over the π‐conjugated benzofuran–chalcone system, whereas the LUMO extends over the enone bridge and partially over the aryl substituent. This electronic redistribution indicates that the dominant absorption band is primarily of π→π* character with partial intramolecular charge–transfer contribution. In contrast, the n→π* transition associated with the carbonyl lone pair is expected to be weaker, which explains why it remains masked by the intense π→π* transition.

The structural rigidity and extended π‐conjugation of the benzofuran–aryl system promote electronic delocalization, leading to relatively low‐energy π→π* transitions and consistent spectral behavior across different solvents.

### 3.2 Fluorescence Emission Analysis

The fluorescence of molecules **4a‐c** in all previously used solvents (10^−5 ^M) was recorded at room temperature.

#### 3.2.1 Steady‐State Emission Spectra

**FIGURE 3:**
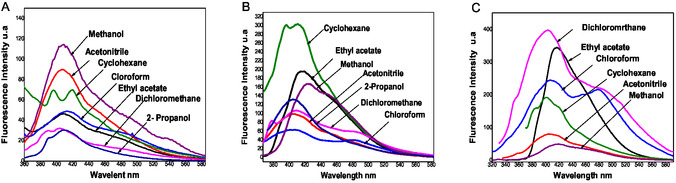
(A–C) Emission spectrum of all compounds (A) **4a** , (B) **4b,** and (C) **4c** in different solvents.

For the three molecules, the shape and position of the emission spectra were independent of the excitation wavelength, confirming that only one species emits in each solution (Figure 3).

For example, the fluorescence emission spectrum of compound **4a** shows an emission maximum at 408 nm in acetonitrile and 409 nm in chloroform. Compound **4b** exhibits emission at 407 nm in dichloromethane, 323 nm in cyclohexane, and 409 nm in chloroform.

All the values of the wavelengths corresponding to the maxima of the fluorescence emission bands of the investigated molecules at 293 K are given in Table 1. It is observed that these molecules do not demonstrate a significant bathochromic shift when changing from polar to nonpolar solvents. This can be explained by the high structural rigidity of the three furochromen derivatives. The rigid conformation limits geometric relaxation between the ground and excited states, thereby reducing the stabilization of the excited state by the solvent. The emission peaks are still present within a similar range for all compounds.

**TABLE 1:** Spectroscopic and photophysical features of the derivatives of these compounds **4(a–c)** in different solvents.

Compounds	Solvent	***λ***_**abs**_, **nm**	***ε*, M** ^**−1 **^ **cm** ^**−1**^	***λ***_**ex**_, **nm**	***λ***_**em**_ **, nm**	***ν***_**a**_ ** − ** ***ν***_**f**_, **cm** ^**−1**^	Ф_f_
**4a**	Acetonitrile	353	28 000	329	408	3819	0.058
	Methanol	355	20 000	300	410	3779	0.107
	Ethyl acetate	352	16 000	310	408	3899	0.057
	CHCl_3_	352	32 000	320	409	3959	0.038
	2‐Propanol	356	17 000	350	405	3759	0.023
	CH_2_Cl_2_	353	28 000	323	407	3819	0.018
	Cyclohexane	350	10 000	310	408	4062	0.154
**4b**	Acetonitrile	330	93 000	314	410	5913	0.021
	Methanol	325	30 000	300	420	6311	0.088
	Ethyl acetate	328	103 000	330	410	6098	0.027
	CHCl_3_	325	44 000	300	409	6319	0.037
	2‐Propanol	325	11 000	327	405	6078	0,165
	CH_2_Cl_2_	332	22 000	340	407	6199	0.091
	Cyclohexane	322	10 000	323	408	6546	0.490
**4c**	Acetonitrile	333	118 000	324	404	5277	0.013
	Methanol	341	23 000	300	419	5459	0.037
	Ethyl acetate	335	94 000	330	417	5870	0.050
	CHCl_3_	328	50 000	310	410	6097	0.159
	2‐Propanol	328	30 000	—	—	—	—
	CH_2_Cl_2_	327	30 000	320	403	5767	0.316
	Cyclohexane	324	21 000	330	402	5989	0.140

*Note:* ***λ***_**abs**_: Absorbance wavelength; ***ε***: corresponding molar extinction coefficient; ***λ***_**ex**_: maximum excitation wavelength; ***λ***_**em**_: maximum emission wavelength and shoulders; ***ν***_**a**_ **− *ν***_**f**_: Stokes shift; and **Φ**_**f**_: fluorescence quantum yield with excitation at the maximum absorption wavelength.

### 3.3 Determination of Dipole Moments in the Excited State in Several Solvents by the Solvatochromic Approach

The dipole moment of a molecule in the excited state is identified by the effect of electric field (internal or external) on its spectral bands' position. The solvent dependence of the absorption and fluorescence band maxima is applied to evaluate the excitation‐state dipole moments of distinct molecules. The equations [Equations (1) and (2)] that afford the optimal results in the change of dipole moments of an excited molecule were proposed by A. Kawski and P. Bojarski [25, 26]:

The difference ${\bar{\nu }}_{a}\;-\;{\bar{\nu }}_{f}$ :

(1) $${\bar{\nu }}_{a}\;-\;{\bar{\nu }}_{f}=-S_{1}f(\varepsilon ,n)+\text{const}$$

And the sum ${\bar{\nu }}_{a}\;+\;{\bar{\nu }}_{f}$ :

(2) $${\bar{\nu }}_{a}\;+\;{\bar{\nu }}_{f}=-S_{2}\Phi (\varepsilon ,n)+\text{const}$$

Where:

(3) Φ(ε,n)=f(ε,n) + 2g(n)

And
(4)

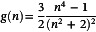




In these Equations, ${\bar{\nu }}_{a}$ and ${\bar{\nu }}_{f}$ are the absorption and fluorescence maxima (cm^−1^), in that order; *n* and *ε* are the refractive index and the dielectric constant of solvents, respectively.

The expressions for the solvent variables *f* (*ε*,*n*) and Ф (*ε*,*n*) are known by A. Kawski and colleagues [27]:

(5) $$f\left(\varepsilon ,n\right)=\frac{2n^{2}\;+\;1}{n^{2}\;+\;2}\left[\frac{\varepsilon \;-\;1}{\varepsilon \;+\;2}\;-\;\frac{n^{2}\;-\;1}{n^{2}\;+\;2}\right]$$

(6)

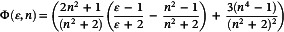




The estimated values of solvent polarity variables f(ε,n) and Φ(ε,n) are given in the Table 2.

**TABLE 2:** Summary of solvent properties and estimated values of solvent polarity variables f(ε,n) and Φ(ε,n).

No	Solvent	*μ*	*ε*	*n*	f(ε,n)	Φ(ε,n)
1	Acetonitrile	3.45	37.5	1.344	0.863	1.331
2	Ethyl acetate	1.88	6.02	1.372	0.489	0.996
3	Methanol	1.70	32.7	1.328	0.855	1.302
4	2‐Propanol	1.66	18.3	1.377	0.765	1.278
5	Dichloromethane	1.55	8.93	1.424	0.596	0.581
6	Chloroform	1.15	4.81	1.446	0.371	0.972
7	Cyclohexane	0.00	2.02	1.426	−0.003	0.575

From Equations (1) and (2), the expressions for slopes *S*_1_ and *S*_2_ are defined by:



(7)

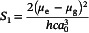

(8) $$S_{2}=\frac{2(\mu_{e}^{2}\;-\;\mu_{g}^{2})}{hca_{0}^{3}}$$

Where *μ*_g_ and *μ*_e_ are ground‐ and excited‐state dipole moments of the solute molecule. The symbols h and c are Planck's constant and the light velocity in vacuum, respectively. $a_{0}$ is the Onsager cavity radius of the solute molecule, and the values were calculated from the molecular volume of dye molecules according to Suppan's equation [27, 28]:$a_{0}={\left(3M/4\pi \delta N\right)}^{1/3}$. Where δ is the density of the solute molecule. *M* is the molecular weight of solute, and *N* is Avogadro's number.

The ground‐ and excited‐state dipole moments are estimated by means of the Equations (9) – (11). Based on Equations (7) and (8), and assuming that the symmetry of the investigated solute molecules remains unchanged upon electronic transition and the ground and excited‐state dipole moments are parallel, one obtains:

(9) $$\mu_{g}=\frac{\left|S_{2}\;-\;S_{1}\right|}{2}{\left(\frac{hca_{0}^{3}}{2S_{1}}\right)}^{1/2}$$

(10) $$\mu_{e}=\frac{\left|S_{2}\;+\;S_{1}\right|}{2}{\left(\frac{hca_{0}^{3}}{2S_{1}}\right)}^{1/2}$$

(11) $$\frac{\mu_{e}}{\mu_{g}}=\frac{\left|S_{2}\;+\;S_{1}\right|}{\left|S_{2}\;-\;S_{1}\right|}\mathrm{Slope}S2>\mathrm{Slope}S1$$

The slopes $S_{1}$ and $S_{2}$ were graphically established by plotting Stokes shifts ${\bar{\nu }}_{a}\;-\;{\bar{\nu }}_{f}$ and ${\bar{\nu }}_{a}\;+\;{\bar{\nu }}_{f}$ versus the bulk solvent polarity functions f(ε,n) and Φ(ε,n) for each solvent, respectively.

Solvent characteristics and polarity results *F* (*ε*,*n*) and Ф (*ε*,*n*) are presented in Table 3. The absorption and emission maxima. Stokes shift ${\bar{\nu }}_{a}\;-\;{\bar{\nu }}_{f}$ and arithmetic mean of Stokes shift ${\bar{\nu }}_{a}\;+\;{\bar{\nu }}_{f}$ for all the molecules in different solvents, and typical absorption and emission spectra of the molecules are given in Table 2.

**TABLE 3:** Statistical procedure of the correlations of solvent spectral shifts 4 (a–c).

Compound	Slope	**Intercept cm** ^**−1**^	Correlation coefficient	Number of data
Equation (1) correlation				
**4a**	334.33229	4058.77574	0.96	6
**4b**	486.17637	6487.93778	0.75	6
**4c**	823.6667	6178.62969	0.85	6
Equation (2) correlation				
**4a**	476.85478	53 267.2353	0.70	6
**4b**	1511.2999	56 387.6584	0.85	6
**4c**	2150.36011	56 699.7259	0.74	6

**FIGURE 4:**
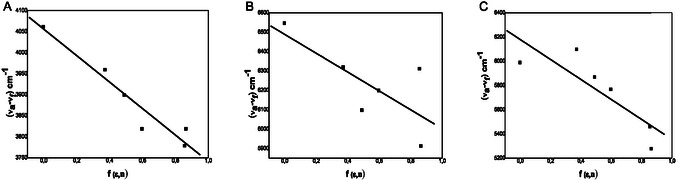
(A–C) The variation of Stokes shift with f(ε,n) by employing Equation (1) in different solvents: (A) **4a** . (B) **4b** and (C) **4c** .

**FIGURE 5:**
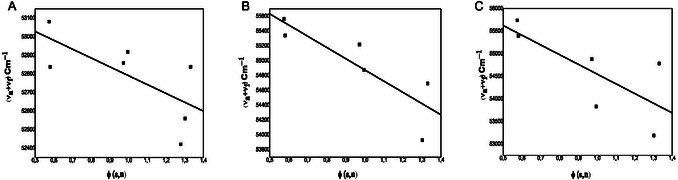
(A–C) The difference of arithmetic sum of Stokes shift with Φ(ε,n) using Equation (2) in different solvents: (A) **4a** . (B) **4b** and (C) **4c** .

The Figure 4 A–C and the Figure 5 A–C display the graph of $\left({\bar{\nu }}_{a}-{\bar{\nu }}_{f}\right)$ versus *f* (*ε*,*n*) and $\left({\bar{\nu }}_{a}+{\bar{\nu }}_{f}\right)$ versus Ф (*ε*,*n*) and the slopes *S*_1_ and *S*_2_ are measured from these graphs, respectively.

The correlation coefficients, slopes, and intercepts of the fitted lines are given in Table 4. Good correlation coefficient is acquired for all cases. The excited‐state dipole moments *μ*_e_ of the three molecules have been calculated by computing the values of *μ*_g_ obtained by Equations (9), (10). The results are given in Table 4. The elevated values of *μ*_e_ and *μ*_g_ and the variation in the dipole moment for all compounds may be related in terms of their possible resonance structures. The molecules **4(a–c)** do not generate a considerable change in the π electron mobility. Upon excitation, the chloro or methyl group becomes a strong electron donor. This describes the higher value of dipole moment both in the excited state and during the charge transfer.

**TABLE 4:** Dipole moments in the ground and excited states and correlation factor (*r*) of (2–9).

Molecules	Radius, A°	*μ*_**g,**_ **D** a	*μ*_**e,**_ **D** b	Δ*μ* **, D** c	*μ*_**e**_ **/** *μ*_**g**_ d
**4a**	4.7908	1.164	6.629	5.465	5.695
**4b**	4.6478	5.26	10.249	4.989	1.948
**4c**	4.7201	4.209	9.434	5.225	2.241

*Note:* Debye = 3.33564 × 10−30C.m = 10−18esuC.m.

a The experimental ground‐state dipole moment calculated from the Equation (9).

b The experimental excited‐state dipole moment calculated from the Equation (10).

c The dipole moment change calculated by Kawski and coworkers Equation (7).

d The ratio of *μ*_e_ and *μ*_g_ is calculated from Equation (11).

Knowledge of the molecular orbitals (MOs) of compounds **4a‐c** and their associated energies enables us to understand chemical reactivity, know their electronic structure, and predict their geometry [28]. Two molecular orbitals are distinguished. HOMO (Highest Occupied Molecular Orbital) reflects the electron‐donating (nucleophilic) character of the compound. The higher the energy of this orbital, the greater the molecule yields electrons. The LUMO indicates the electro‐acceptor (electrophilic) character of the compound. The declined the energy of this OM, the more readily the molecule accepts electrons.

The energy difference between these orbitals (Δ*E* = *E*_LUMO_ – *E*_HOMO_) is a key parameter governing molecular stability and reactivity.

The calculated HOMO and LUMO energies for compounds 4a–c are listed in Table 5.

**TABLE 5:** Energies of frontier molecular orbitals.

Structure	**4a**	**4b**	**4c**
**HOMO, eV**	−5.415	−5.494	−5.571
**LUMO, eV**	−1.952	−2.130	−2.310
**Gap energy, eV**	3.463	3.364	3.261

The obtained HOMO–LUMO energy gaps follow the order: 4a (3.463 eV) > 4b (3.364 eV) > 4c (3.261 eV) [29].

Compound **4a**, bearing an electron‐donating methoxy substituent, exhibits the largest energy gap, indicating higher kinetic stability and lower chemical reactivity [30, 31]. In contrast, compound **4c**, containing an electron‐withdrawing chlorine substituent, presents the smallest energy gap (3.261 eV).

A smaller energy gap is associated with increased molecular softness, enhanced polarizability, and greater chemical reactivity. This suggests that compound **4c** can more easily participate in charge–transfer interactions with biological targets such as proteins or nucleic acids.

Furthermore, the progressively decreasing LUMO energies from 4a (−1.952 eV) to 4c (−2.310 eV) indicate improved electron‐accepting ability in the chlorinated derivative. This enhanced electrophilic character may facilitate stronger binding interactions with nucleophilic residues in biological systems, potentially contributing to its superior antiproliferative activity.

The correlation between the reduced HOMO–LUMO gap and enhanced cytotoxic activity suggests that electronic factors play a significant role in modulating the biological behavior of these derivatives. In particular, the higher reactivity and electron‐accepting ability of compound **4c** may facilitate its interaction with intracellular biomolecules, thereby explaining its superior antiproliferative effect.

**FIGURE 6:**
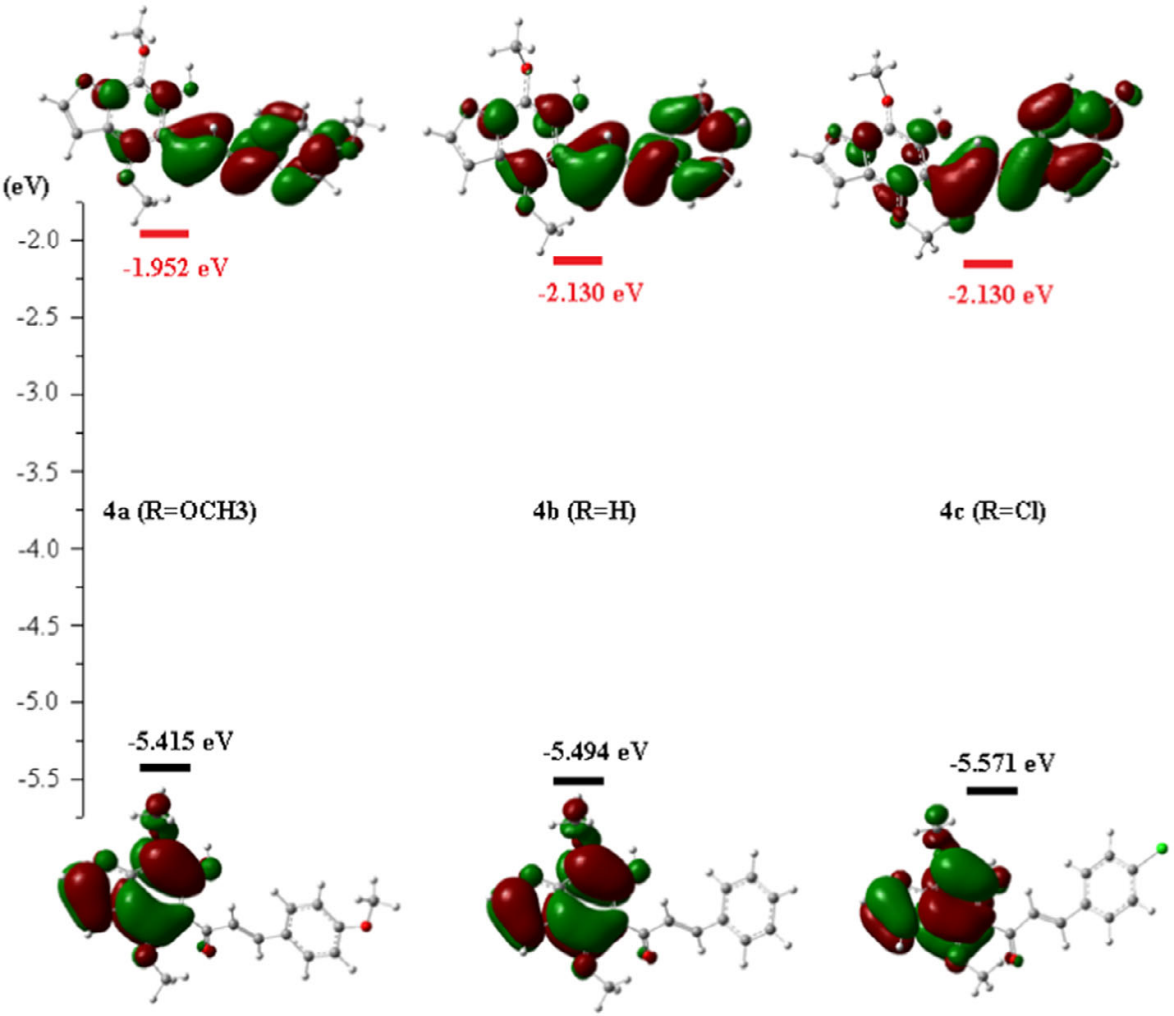
The frontier molecular orbital of furochromene derivative structures (**4a**, **4b,** and **4c)**.

Analysis of the molecular orbitals of the furochromene derivatives shown in Figure 6 revealed similarities in the shape of the molecular orbitals, such that the highest occupied level is located on the benzofuran moiety, while the excited frontier orbital is located on the majority of the skeleton of the compounds studied. This highlights that the energy and spatial position of HOMO and LUMO are important for understanding electron transfer and that the transitions allowed between these different electronic energy levels are characteristic of the UV‐visible absorption spectrum of systems.

To conclude, the dipole moments in the ground and excited states indicate a limited electronic redistribution upon excitation. Consequently, the electronic transition energy remains largely insensitive to solvent polarity, which explains the minimal solvatochromic shifts observed experimentally. Moreover, the excited‐state dipole moment values (*μ*_e_) ranging from 6.6 to 10.2 D and the changes Δ*μ* of about 5 D for the three derivatives confirm a similar electronic behavior across the series. This moderate electronic stability, coupled with structural rigidity, prevents the formation of an excited state with a strong charge–transfer character. These results are also consistent with the theoretical data presented in the frontier molecular orbital energy table, where the R = OCH_3_ derivative exhibits a wide HOMO‐LUMO gap (3.463 eV) compared to the other derivatives (R = H: 3.364 eV; R = Cl: 3.261 eV). This large gap indicates high kinetic stability and low chemical reactivity, reinforcing the idea that molecular rigidity limits electronic variations upon excitation and explains the absence of significant bathochromic shifts with respect to solvent polarity.

### 3.4 Antiproliferative Activity of Synthesized Furochromene Derivatives against Human Tumor Cells

**FIGURE 7:**
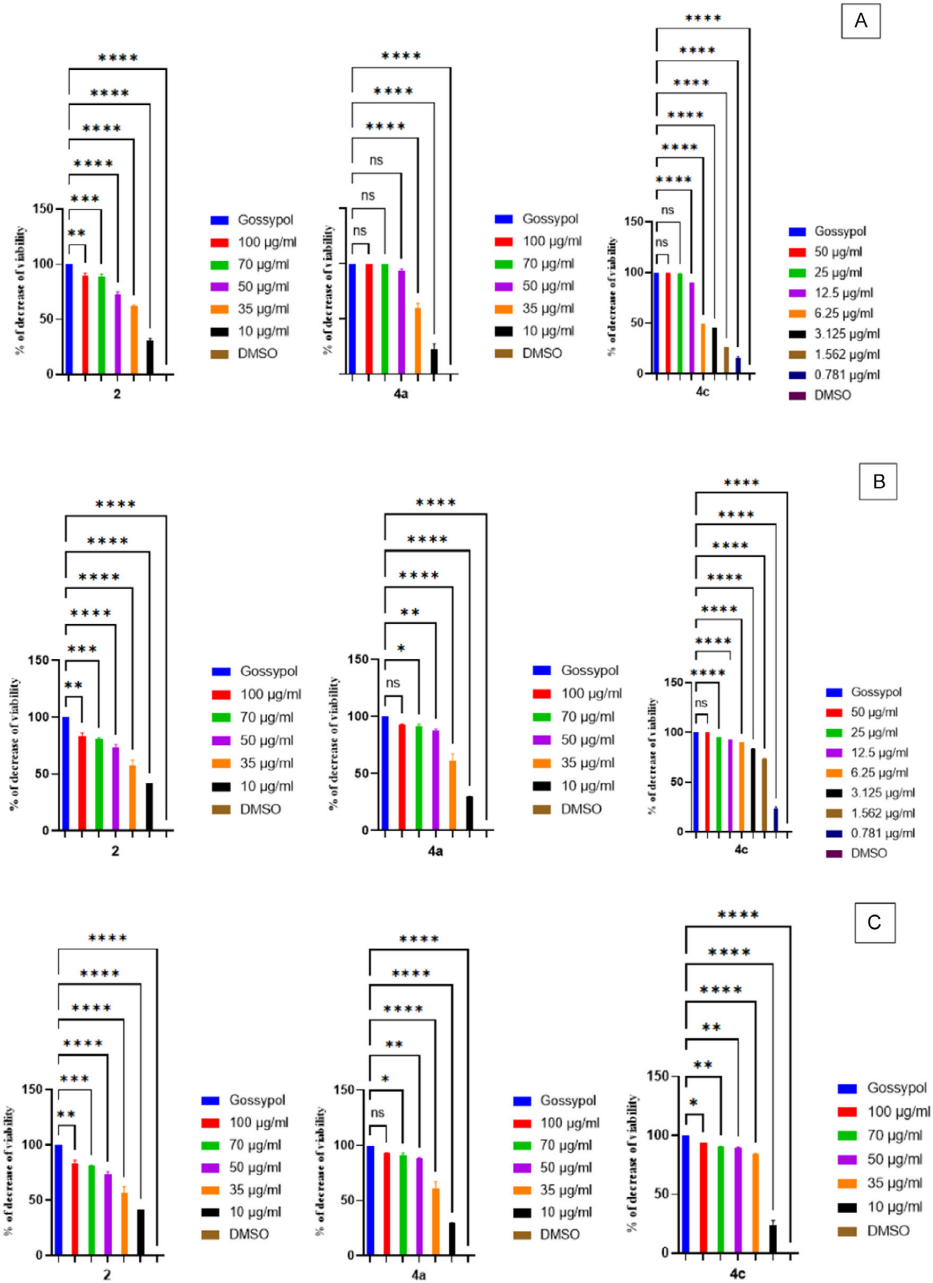
Effects of compounds 2, 4a, and 4c on viability of: HCT‐116 (A), HepG2 (B), and A549 (C) cells. Control corresponds to solvent‐treated cells. **ns**: not significant, *p* < 0.05 (*), *p* < 0.01 (**), *p* < 0.001 (***), *p* < 0.0001 (****).

Tumor cells were exposed to five different concentrations of the compounds: 100, 70, 50, 35, and 10 µM, for a duration of 24 h. Gossypol, a known pro‐apoptotic compound, was used as a positive control, while 0.5% DMSO served as a negative (solvent) control. The study of the antiproliferative effects of our compounds **2** , **4a** , and **4c** revealed that these molecules possess significant cytotoxic potential. The antiproliferative assay results revealed that the **2** , **4a** , and **4c** compounds reduced the viability of A549, HepG2, and HCT‐116 tumor cells to varying degrees, as shown in Figure 7. The cell viability percentage decreased with higher **2** , **4a** , **4c** compound concentrations and extended incubation times. Among them, **4c** exhibited the most potent cytotoxic effect, completely inhibiting the viability of A549 and HepG2 cells (100%) and reducing HCT‐116 cell viability by 98.55% at a concentration of 100 µM.

**2** and **4a** also demonstrated significant antiproliferative activity, particularly against A549 cells. **2** decreased the viability of A549, HepG2, and HCT‐116 cells by 90.7%, 88.12%, and 91.44%, respectively, at 100 µM. Similarly, 4a reduced A549 cell viability by 92.1%, HepG2 cell viability by 99.32%, and completely inhibited HCT‐116 cell viability (100%) at the same concentration.

The IC50 values were derived from the cell viability reduction graph after 24 h of incubation and are summarized in Table 6. The treatment of A549 cells with **4c** for 24 h gave a low IC50 of 4.605 ± 0.485 µM, indicating the highest efficacy.

**TABLE 6:** IC_50_ values of synthesized furochromene derivatives against the studied human cancer cell lines.

**IC**_**50**_ **µg/ml**	**Compound** **2** **(C2)**	**Compound** **4a** **(C4a)**	**Compound** **4c** **(C4c)**	Statistical records (*p* value)
A549	27.77 ± 0.725	11.06 ± 0.27	4.605 ± 0.485	C2‐C4a: *** C2‐C4b: **** C4a‐C4b: **
HepG2	16.64 ± 0.86	17.64 ± 0.715	17.21 ± 0.6	C2‐C4a: ns C2‐C4b: ns C4a‐C4b: ns
HC116	21.02 ± 0.81	33.26 ± 0.48	7.105 ± 0.835	C2‐C4a: *** C2‐C4b: *** C4a‐C4b: ****

*Note:* IC_50_ values (µg/mL) of compounds 2, 4a, and 4c against human cancer cell lines A549, HepG2, and HCT‐116 are expressed as mean ± SD from three independent experiments (*n* = 3). Statistical comparisons were performed using one‐way ANOVA followed by Tukey's post hoc test. Significance symbols: ns, not significant; *p < 0.05; **p < 0.01; ***p < 0.001; ****p < 0.0001.

## 4 Discussion

Furochromenes are heterocyclic compounds with a fused structure combining a furan and a chromene core. This configuration provides them with conformational rigidity and a conjugated system that promotes interactions with biological targets, particularly regulatory proteins involved in the cell cycle and apoptosis [32]. The chromene core can participate in π‐π interactions with biological macromolecules, while functional groups on the furan ring can modulate solubility, lipophilicity, and chemical reactivity, thereby influencing their bioavailability and anticancer selectivity [33]. Several studies published in the literature reveal that furochromenes exhibit anticancer activity that is modulated by the type and position of their substituents. For example, derivatives with a chlorine group (Cl) show higher activity than those containing a methoxy group (OCH_3_) [7], which is explained by the electronic effect of chlorine that enhances interaction with the cancer target. Conversely, another study highlights that furochromenes carrying methoxy groups can also improve anticancer activity by modulating their ability to interact with DNA or specific enzymes [3]. These findings indicate that the effectiveness of substituents depends not only on their chemical nature but also on their position on the furochromene scaffold, showing the importance of precise structural tuning to optimize biological activity.

The study conducted on the derivatives 2, **4a**, and **4c** is based on their distinct electronic effects, allowing a rational exploration of the influence of electron‐donating or electron‐withdrawing groups on the physicochemical and biological properties of the compounds. The methoxy group (OCH_3_), with its electron‐donating mesomeric effect, can increase the electron density and modify the dipole moment as well as the fluorescence properties, while the hydrogen atom (H) serves as a neutral reference to compare the variations induced by the substituents. Chlorine (Cl), on the other hand, exerts an electron‐withdrawing inductive effect and a mesomeric donating effect primarily, potentially altering polarity, electronic distribution, and interactions with biological targets.

This comparative approach thus enables the establishment of a correlation between electronic effects, photophysical behavior, and anticancer activity, providing a mechanistic basis for interpreting the results and guiding the design of these compounds, which showed significant inhibition of cell proliferation in the human cancer cell lines A549, HepG2, and HCT116.

Among these compounds, **4c** exhibited the highest cytotoxicity, suggesting stronger interactions with biological targets. Structure‐activity relationship (SAR) analysis indicates that the presence of a chlorine atom in **4c** could explain its enhanced efficacy. On one hand, chlorine is an electron‐withdrawing group, altering the electronic distribution of the furochromene core and strengthening its interaction with proteins involved in cell proliferation [33]. On the other hand, its increased lipophilicity may enhance membrane permeability, thereby promoting intracellular accumulation of the compound. Finally, some halogenated derivatives are known to induce oxidative stress through the production of reactive oxygen species (ROS), a potential mechanism that could contribute to the cytotoxic effect of **4c** [34]. Similar studies have demonstrated that furochromene derivatives can induce apoptosis, inhibit kinases, or block cell cycle progression in cancer cell lines such as A549 and HepG2. For instance, one study reported that hybrid phosphomolybdenum compounds exhibited potent inhibitory activity against MCF‐7, A549, and HepG2 cells, with IC50 values of 33.79 µM, 25.17 µM, and 32.11 µM, respectively [35]. Comparatively, 4c demonstrates competitive cytotoxicity, making it a promising candidate for further investigations. Cytotoxicity in this study was evaluated on human cancer cell lines, with future investigations planned to assess selectivity toward noncancerous cell lines. All experiments were performed in triplicate (*n* = 3), and statistical analyses were conducted using one‐way ANOVA followed by appropriate post hoc tests, ensuring reproducibility and reliability of the observed trends. To further elucidate the mechanisms underlying 4c's efficacy, additional studies are warranted, including apoptosis and cell cycle analyses, ROS production assays, gene expression studies, and molecular modeling. Complementary in vivo investigations with larger experimental replicates will be critical to confirm and expand upon these findings.

## 5 Conclusion

The synthesized furochromene derivatives **2** , **4** **(a–c)** exhibited notable photophysical and biological properties. Their fluorescence behavior in various solvents, along with their significant Stokes shifts and solvatochromic responses, supports their utility as potential fluorescent probes. Furthermore, the electronic structures and dipole moment analyses indicate favorable molecular characteristics for interactions with biological targets. The antiproliferative evaluations revealed that compound **4c** , in particular, displays strong cytotoxic activity across multiple cancer cell lines, especially A549 and HepG2. These results suggest that furochromene‐based compounds may serve as promising leads for the development of novel anticancer agents with intrinsic fluorescent properties. Future work will focus on elucidating the mechanism of action and optimizing these molecules for in vivo studies.

## Author Contributions

**Amal Rabahi**: writing – original draft preparation, synthesis of the studied compounds, and conceptualization. **Mustapha Mounir Bouhenna**: writing, realization of cytotoxic activity assays, and review & editing, project administration. **Hasnia Abdeldjebar**: writing – review & editing, and formal analysis. **Massinissa Baymout**: UV–Visible absorption and fluorescence emission analyses. **Malika Makhloufi‐Chebli**: writing – review & editing. **Walter Luyten**: cytotoxicity assays funding; writing – review & editing, **Anis Ahmad Chaudhary**: writing—review and editing. **Walid Elfalleh**: data curation, project administration. **Hamdi Bendif, Stefania Garzoli**: writing—review and editing, supervision, project administration.

## Funding

This work was supported and funded by the Deanship of Scientific Research at Imam Mohammad Ibn Saud Islamic University (IMSIU) (grant number IMSIU‐DDRSP2601).

## Conflicts of Interest

The authors declare no conflicts of interest.

## Data Availability

All the data in the article are available from the corresponding author upon reasonable request.
